# Galanin-expression and galanin-dependent sensory neurons are not required for itch

**DOI:** 10.1186/1744-8069-8-87

**Published:** 2012-12-05

**Authors:** Fiona E Holmes, Penny Vanderplank, David Wynick

**Affiliations:** 1 Schools of Physiology and Pharmacology and Clinical Sciences, University of Bristol, Bristol, BS8 1TD, United Kingdom

**Keywords:** Itch, Galanin, Pain

## Abstract

**Background:**

Galanin is a key modulator of nociception, and it is also required for the developmental survival of a subset of C-fibre sensory neurons which are critical to the mediation of neuropathic and inflammatory pain. However, the potential modulatory roles played by galanin, or the galanin-dependent neurons, in pruritoceptive mechanisms underlying the sensation of itch have not been investigated.

**Findings:**

Here we report that mice carrying a loss-of-function mutation in the galanin gene (Gal-KO) show no differences in spontaneous behavioural itch responses compared to wild-type (WT) controls. Similarly, the responses to a range of pruritogens are not significantly different between the two genotypes.

**Conclusions:**

These results suggest that neither galanin expression, nor the galanin-dependent subpopulation of sensory neurons is required for itch-related behaviours.

## Findings

### Introduction

Itch and pain are complex unpleasant sensory experiences that provoke aversive yet protective responses. Like pain, itch may become a chronic pathological condition. However, the relationship between the neuronal circuits that mediate itch and pain is unclear. Emerging evidence has identified specific subpopulations of peptidergic and non-peptidergic peripheral sensory C-fibre neurons, some of which constitute a proportion of the nociceptive neuronal population, that appear to mediate the sensation of itch [1-3]. Several neuropeptides including gastrin-related peptide (GRP) [4], calcitonin gene-related peptide [5] and substance P [6] have been shown to modulate the neurotransmission of itch, in addition to glutamate [7]. Further, genetic ablation of MrgprD neurons which account for approximately 90% of cutaneous non-peptidergic neurons does not appear to affect behavioural responses to several pruritogens [8], implying an important role for the peptidergic population in itch signalling.

Given the similarities between chronic itch and pain [9], and apparently similar subpopulations of sensory neurons thought to mediate these functional pathways, the two circuits may be related, if not overlapping. Targeted disruption of the GRP and TLR7 receptors and the chromosomal locus encoding 12 Mrgpr genes all attenuate itch induced by various pruritogens, but have no significant effect on nociceptive, neuropathic or inflammatory pain behaviours [4,10,11]. In contrast, abolition of VGLUT2-dependent synaptic glutamate release from nociceptors greatly enhances chronic itching and significantly reduces neuropathic and inflammatory pain behaviours [12].

Taken together, the above data provide evidence that some pruritogens are dependent on subsets of peptidergic C-fibre neurons for their effects, though whether these circuits overlap with, or are distinct from, those that mediate neuropathic pain is still uncertain. To address this question, we studied mice with a targeted deletion of the neuropeptide galanin (Gal-KO) which lose a subset of small diameter unmyelinated peptidergic sensory neurons shortly after birth [13].

Galanin is expressed during development by the majority of dorsal root ganglion (DRG) neurons, from E13 until shortly after birth [14], after which it is down-regulated. In the adult, it is expressed in about 5% of, predominantly C-fibre, DRG neurons [15]. Higher levels are expressed in primary afferent terminals and interneurons in the spinal cord dorsal horn, and in several brain areas important for pain processing [16]. After nerve injury, galanin in the DRG increases significantly in about 30% of sensory neurons, and moderately increases in the dorsal horn [17]. Intrathecal administration of low dose galanin has modest facilitatory effects on nociception, whereas higher doses cause marked inhibition. In models of nerve injury when endogenous levels of galanin are high, the neuropeptide inhibits pain behaviour [18,19].

Galanin also plays a developmental cell survival role in the DRG. In the absence of galanin, a subset of peptidergic C-fibre neurons (13% of all DRG neurons) are lost by apoptosis shortly after birth [13]. These neurons seem to be pivotal to the development of neuropathic and inflammatory pain behaviours, which are absent or greatly diminished in Gal-KO mice [20-22].

In the present study we asked whether itch behaviours to a variety of pruritogens known to act on different subpopulations of primary sensory neurons are altered in the Gal-KO animals.

### Methods

#### Mice

Gal-KO mice were generated as previously described [13] and maintained on the inbred 129OlaHsd strain with identical strain-matched wild-type (WT) controls. 10–12 weeks old male mice were used for all experiments. All work performed conformed to United Kingdom Home Office legislation (Scientific Procedures Act 1986). Procedures were conducted in accordance with UK Home Office principles of laboratory animal care.

#### Itch-related behaviours

Mice were habituated to the testing environment and subsequent behaviour recorded by video camera. Basal spontaneous scratching behaviour was monitored then mice were injected intradermally, using a BD Microfine 0.3 ml syringe with 29G needle, with 50 μl PBS to determine scratching responses to vehicle. Subsequently, the following pruritogens were injected in a volume of 50 μl: 100 μg (650 nmol) compound 48/80; 100 μg (900 nmol) histamine; 25 ng (10 pmol) endothelin-1; 60 μg (196 nmol) α-methyl-serotonin; 100 μg (152 nmol) SLIGRL-NH_2_; 200 μg (388 nmol) chloroquine; n = 8 for each group. Numbers of scratching bouts were scored in 5 min increments over the 30 or 60 min period by an observer blind to the genotype tested. A scratching bout was defined as one or more rapid hind paw movements directed at the injection site, ending with the mouse placing its hind paw back on the floor.

#### Cheek model of itch or pain

Basal wiping and scratching behaviour was monitored for 30 min prior to cheek injection of 20 μg capsaicin or vehicle (10% ethanol, 10% Tween 80 in PBS) in 20 μl. Numbers of forelimb wipes or hindlimb scratches directed at the injection site were counted in 5 min increments over 20 min; n = 8 for the vehicle-injected group and n = 16 for the capsaicin-injected group.

#### Data analysis

Unpaired *t-*tests were used to compare behavioural data from WT and Gal-KOs. Data are presented as mean ± SEM. *P* < 0.05 was considered significant.

### Results

The scratching reflex in rodents in response to intradermal or subcutaneous injection of a pruritogen is a well characterised behavioural correlate of itch [23]. Pruritogens may act directly on sensory primary afferents via specific receptors to activate itch and/or indirectly via non-neuronal cells in the skin. In this study the number of spontaneous scratch bouts, as well as the number elicited by various pruritogens, were measured in strain-, sex- and age-matched WT and Gal-KO mice.

Gal-KO and WT mice both showed minimal spontaneous scratching behaviours. Similarly, no scratching behaviours in either genotype were observed following a vehicle injection of PBS.

**Figure 1:**
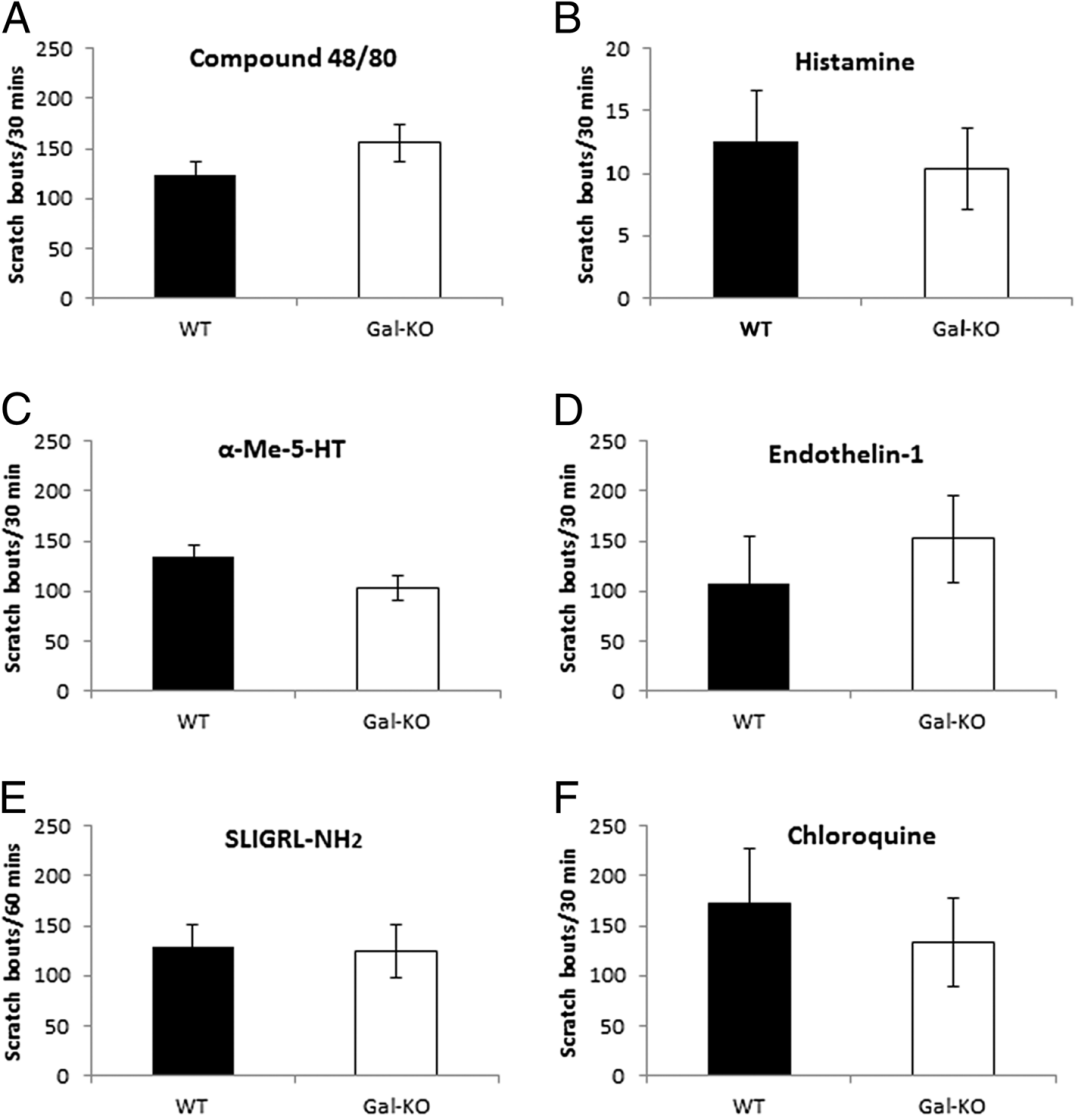
**Pruritogen**-**induced itch**-**related behaviour in WT and Gal-KO mice.** Numbers of scratching bouts were measured in WT and Gal-KO mice after intradermal injection of: (**A**) compound 48/80; (**B**) histamine; (**C**) α-methyl serotonin; (**D**) endothelin-1; (**E**) SLIGRL-NH_2_ and (**F**) chloroquine. Results are presented as means ± SEM.

Compound 48/80 promotes mast cell degranulation and thus predominantly elicits itch indirectly via the release of several pruritogens from mast cells, mainly histamine. Over a 30 min period, WT mice exhibited 124 ± 13 scratch bouts and Gal-KO mice 156 ± 19 (*p* = 0.193) (Figure 1A).

Histamine acts via H_1_ and H_4_ receptors, both expressed by C-fibre neurons in the DRG. Histamine elicited very weak itch responses of 13 ± 4 and 10 ± 3 scratch bouts in WT and Gal-KO mice, respectively (*p* = 0.683) (Figure 1B).

α-Methyl-5-hydroxytryptamine, a serotonin receptor agonist, targets the 5-HT_2_ metabotropic receptor. It elicited robust scratching in WT mice (135 ± 11 bouts). Less scratching was seen in Gal-KO mice (103 ± 12 bouts), but the difference was not statistically significant (*p* = 0.07) (Figure 1C).

Endothelin-1 induces itch via activation of ET_A_ receptors. It induced similar scratching behaviour in WT (108 ± 45) and Gal-KO mice (153 ± 43; *p* = 0.492) (Figure 1D).

SLIGRL-NH_2_ is a PAR-2 agonist, but may induce itch through activation of MrgprC11 [24] in the non-peptidergic population of C-fibre neurons. SLIGRL-NH_2_ induced comparable scratching responses in both WT (129 ± 22) and Gal-KO mice (124 ± 27; *p* = 0.894) (Figure 1E).

Chloroquine also activates a histamine-independent itch pathway, which has recently been shown to be mediated via activation of MrgprA3 expressed by non-peptidergic C-fibre neurons [25]. Again, there was no significant difference in itch-related behaviours in WT and Gal-KO mice (134 ± 45 *vs* 173 ± 54; *p* = 0.658) (Figure 1F).

Although it is clear that galanin is involved in the modulation of pain, its precise mode of action via its receptors, GalR1 and GalR2, is complex and remains incompletely understood. Most current evidence suggests that galanin has an inhibitory role in nociception, mediated post-synaptically via the hyperpolarisation of GalR1-expressing glutaminergic dorsal horn neurons. Conversely, galanin appears to activate GalR2 pre-synaptically. Both GalR1 and GalR2 mRNA are expressed in partially overlapping populations of primary sensory neurons, whereas there is little GalR2 expression in intrinsic dorsal horn neurons. GalR2 activation has both facilitatory and inhibitory effects on neurotransmission, depending on the local concentration of galanin. However, in models of neuronal damage, when endogenous levels of galanin are increased, its inhibitory role is predominant. As well as being expressed in DRG neurons, galanin is also present in a small population of inhibitory interneurons, but their role in the modulation of acute or chronic pain is unknown. Determining the precise distribution of GalR1 and GalR2 has been hampered by a lack of specific antibodies. Furthermore, a paucity of receptor-specific agonist and antagonists had made the elucidation of the role of galanin and its receptors in pain and itch circuitry difficult.

Current data suggest that pain and itch circuits are antagonistic: Pain appears to suppress itch and inhibition of pain evokes itch. Therefore, on the one hand, it might be expected that a lack of the neuromodulatory actions of galanin may reduce itch, as might a loss of a subset of C-fibre neurons, if they express molecules required for mediating pruritic behaviours. Conversely, the loss of a subset of galanin-dependent nociceptors in the Gal-KO that mediate chronic neuropathic and inflammatory pain behaviours, may enhance itch. Lastly, it is possible that the neuromodulatory and survival roles played by galanin may have opposing effects on itch and thus mask a subtle phenotype in the Gal-KO mice.

**Figure 2:**
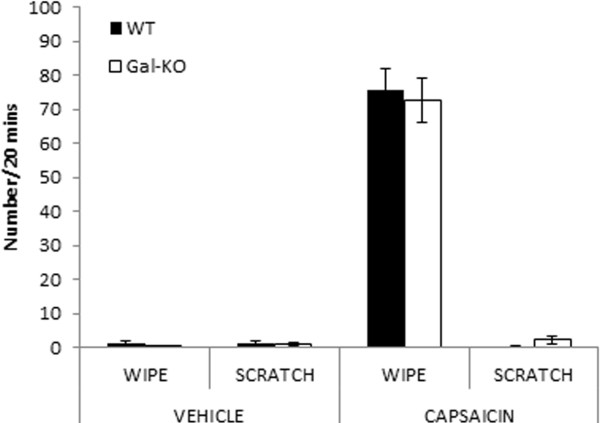
**Capsaicin**-**induced pain or itch in WT and Gal-KO mice.** Pain (forelimb wiping) and itch (hindpaw scratching)-related behaviours were measured after intradermal cheek injection of vehicle or capsaicin. Results are presented as means ± SEM.

To further study the role of the galanin-dependent sensory neuron population in the modulation of itch, we used the cheek model of itch/pain [26]. This model allows the behavioural differentiation of itch and pain; substances which elicit pain cause enhanced forepaw wiping of the injection site, whereas substances which elicit itch cause hindpaw scratching. Cheek injection of capsaicin caused wiping behaviour but no scratching behaviour. There was no significant difference between WT and Gal-KO mice (*p* = 0.743) (Figure 2). Previously we have shown that Gal-KOs have deficits in pain-like behaviour in response to intraplantar injection of formalin or carrageenan [22]. It is likely that these chronic inflammatory agents act through a range of different mechanisms to induce pain-like behavioural responses. For example, capsaicin has a short-acting effect when injected in the cheek, acting through TRPV1, whereas the first phase of the formalin response is mediated by direct activation of nociceptors via TRPA1 and the second phase reflects central sensitisation. Therefore different injection sites, behavioural outcomes and modes of action of the inflammatory agents may explain the absence of a difference in acute nociception in the Gal-KO and WT mice. Further, since the effects of capsaicin are mediated via TRPV1, which is expressed in about half of peptidergic nociceptors, it is also possible that the remaining TRPV1-positive neurons in the Gal-KO are sufficient to induce the maximal wiping response in the cheek model of itch/pain.

In summary, our data does not support a tonic role for galanin in modulating itch in the adult. Similarly, the galanin-dependent population of peptidergic neurons which we have shown to be critical for mediating the full expression of neuropathic and inflammatory pain behaviours appear to be dispensable for itch. These findings are therefore consistent with the hypothesis that itch and neuropathic pain are mediated by distinct and specific signalling pathways.

## Competing interests

The authors declare that they have no competing interests.

## Authors’ contributions

FEH and DW conceived and designed the study. FEH generated the data using mice bred and genotyped by PV. FEH and DW wrote the manuscript, which was read and approved by all authors.
